# Trichophyton rubrum inhibits Candida albicans filamentation and its gene expression when grown in biofilms in vitro

**DOI:** 10.1590/0074-02760240221

**Published:** 2025-06-27

**Authors:** Níura Madalena Bila, Carolina Orlando Vaso, Jenyffie Araújo Belizário, Mariana M Santoni Biasioli, Ana Marisa Fusco-Almeida, Luis R Martinez, Caroline Barcelos Costa-Orlandi, Maria José Soares Mendes-Giannini

**Affiliations:** 1 Universidade Estadual Paulista, Faculdade de Ciências Farmacêuticas, Departamento de Análises Clínicas, Araraquara, SP, Brasil; 2 Universidade Eduardo Mondlane, Faculdade de Veterinária, Departamento de Sanidade e Saúde Pública, Maputo, Mozambique; 3 Universidade Estadual Paulista, Faculdade de Ciências Farmacêuticas, Departamento de Ciências Biológicas, Araraquara, SP, Brasil; 4 University of Florida College of Dentistry, Department of Oral Biology, Gainesville, FL, United States of America; 5 University of Florida, Center for Translational Research in Neurodegenerative Disease, Gainesville, FL, United States of America; 6 University of Florida, McKnight Brain Institute, Gainesville, FL, United States of America; 7 University of Florida, Center for Immunology and Transplantation, Gainesville, FL, United States of America; 8 University of Florida, Emerging Pathogens Institute, Gainesville, FL, United States of America

**Keywords:** antagonism, mixed biofilms, filamentation, dermatomycoses, gene expression, virulence factors

## Abstract

**BACKGROUND:**

Dermatomycoses are caused by various fungi, including dermatophytes and *Candida* species, which are the most prevalent in isolated or associated forms. A great number of virulence factors expressed by these fungi are important for infection, and biofilm formation leads to the persistence of these infections.

**OBJECTIVES:**

This work aimed to evaluate the dynamics of *Candida albicans* filamentation genes in biofilms formed by *Candida albicans* and *Trichophyton rubrum*.

**METHODS:**

The effect of the supernatants on the biofilms was assessed by XTT reduction assay, confocal microscopy, and gene expression profile analysis by real-time polymerase chain reaction (RT-PCR).

**FINDINGS:**

The supernatants did not reduce the metabolic activities or damage the topography of the monospecies biofilms but caused a reduction in their thickness. The filamentation of *C. albicans* was inhibited when both fungi were cultivated directly. The filamentation genes studied (CPH1, HWP1, and EFG1) were negatively modulated in *C. albicans*.

**MAIN CONCLUSIONS:**

Our findings suggest that the antagonistic relationship shown by *T. rubrum* against *C. albicans* may be attributed to alterations of *C. albicans* filamentous genes.

Dermatomycoses are among the most prevalent mycoses worldwide. These skin infections are mainly caused by *Candida* species and dermatophytes.^1^ ^,^ ^2^ Polymicrobial interactions within multispecies biofilms are among the most frequently reported clinical problems. *Candida* and dermatophytes may form biofilms or microbial communities associated to a solid surface, being a significant virulence factor for these fungi.^3^ ^,^ ^4^ Garcia et al. demonstrated *in vitro* that mixed biofilms composed of *Candida albicans* and *Trichophyton rubrum* showed a predominance of *C. albicans.* ^5^ Interestingly, in this antagonistic relationship, *C. albicans* cells exhibited filamentation inhibition.

The transition of *C. albicans* from planktonic to biofilm growth is accompanied by a complex phenotypic remodeling followed by gene expression changes.^3^ Studies have shown that essential genes control the conversion from yeast to filamentous phase as a hyphal-specific protein (*hwp1*) is related to adhesion.^6^ ^,^ ^7^ Some of these transcription factors, such as CPH1 (transcriptional regulator involved in morphogenesis) and EFG1 (transcriptional regulator related to morphogenesis), have also been shown to be necessary for *C. albicans* biofilm formation.^8^ This study aimed to investigate the direct and indirect interaction between *C. albicans* and *T. rubrum* on their individual growth with particular emphasis on *C. albicans* filamentation and the expression of its filamentation-related genes.

## MATERIALS AND METHODS

*Preparation of monospecies biofilms and mixed biofilms* - Monospecies biofilms of *T. rubrum* (ATCC 28189) and *C. albicans* (SC 5314, also known as ATCC MYA-2876) were prepared as described by Costa-Orlandi et al.^9^ and Pierce et al.,^10^ respectively. *T. rubrum* biofilms were incubated under shaking (150 rpm) at 37ºC for 72 h whereas *C. albicans* biofilms were cultured without shaking at 37ºC for 72 h. Mixed biofilms were prepared for both species in direct form, with a final cell concentration of 1×10^6^ cells/mL, according to Garcia et al.^5^ After *T. rubrum* preadhesion (4 h), the inoculum of *C. albicans* diluted in RPMI 1640 medium was added to the wells, and the 96-well plates (Kasvi) were then incubated at 37ºC for 72 h. The indirect formation of mixed biofilms was performed according to Standar et al.^11^ with modification. For this, a transwell system (Greiner Bio-one, ThinCertTM) with a pore size of 0.4 µm was used. *C. albicans* cells were deposited in the sterile transwell system previously placed in the well containing the *T. rubrum* cells. In this way, *T. rubrum* and *C. albicans* were grown in the same culture medium, interacting chemically only with molecules secreted into the medium but without physical contact. The plates were incubated at 37ºC for 72 h.

*Evaluation of the antibiofilm activity of monospecies supernatants* - Cell-free supernatant was prepared as described by Tan et al.^12^ with minor modifications. Briefly, the supernatants of monospecies biofilms were collected and centrifuged at 3,000 rpm for 10 min. After that, the supernatant was filtered using a 0.2 μM pore size filter (Kasvi). After forming mature monospecies biofilms and removing the culture medium, 100 µL of the cell-free supernatant + 100 µL of RPMI 1640 medium was added. *T. rubrum* biofilms were treated with *C. albicans* supernatant and vice versa and incubated at 37**º**C for 96 h for *T. rubrum* and 24 h for *C. albicans*. The incubation times for each fungal species were standardized according to their cellular metabolism as described elsewhere.^9^ ^,^ ^10^ The metabolic activities of the biofilms were measured by the XTT (2,3-Bis-(2-Methoxy-4-Nitro-5-Sulfophenyl)-2HTetrazolium-5-Carboxanilide) reduction assay (Gibco, Thermo Fisher Scientific, Waltham, MA, USA) and compared with untreated controls.^13^

*Topographic analysis of biofilms by scanning electron microscopy (SEM)* - Biofilms were formed in 24-well plates (Kasvi) and processed for imaging, as described by Costa-Orlandi et al.^9^ Initially, the treated mature biofilms were washed with phosphate-buffered saline (PBS) and fixed with 400 µL of 2.5% glutaraldehyde solution (Sigma-Aldrich) and 400 µL of 4% paraformaldehyde solution for 1 h at 4ºC. Next, the samples were washed and then dehydrated with increasing concentrations of ethyl alcohol from 50% to absolute ethanol and further dried under the same conditions. Before microscopic analysis, the bottom of the plate containing the samples was cut off with a scalpel, mounted in aluminum cylinders with silver (stubs), and placed in a high vacuum evaporator (Denton Vacuum Desk V, Jeol USA) for gold plating. The damage to the topography of the biofilms was analyzed with a Jeol JSM6610LV scanning electron microscope located at the UNESP-Araraquara Dental School, Brazil. ImageJ software was used for image analysis.

*Evaluation of thickness and filamentation inhibition of supernatant-treated and mixed biofilms* - *T. rubrum* cells were stained with a calcofluor white solution (Thermo Fisher Scientific) (100 mg/L) and incubated at 37**º**C for 45 min in the dark. *C. albicans* cells were stained with 5(6)-Carboxyfluorescein diacetate N-succinimidyl ester (CFSE) (Thermo Fisher Scientific) (5 µM) and incubated at 37**º**C for 20 min. Calcofluor white has a high affinity for cell wall polysaccharides such as chitin, which allows for detailed visualization of the fungal structures of *T. rubrum*. CFSE was used to label *C. albicans* due to its ability to covalently bind to amines in intracellular proteins, providing a stable and useful label for tracking cell divisions and monitoring their dynamics in the biofilm. After staining, biofilms (monospecies and dual-species) were performed in 24-well plates covered with glass slides previously sterilized.^13^ Filamentation was determined visually after close and careful observation of the fungal growth. The biofilms were observed in a confocal microscope (Carl Zeiss LSM 800 with Airyscan) with an image capture and processing program (ZEN BLUE 3.2 Software) at the Faculty of Dentistry of UNESP-Araraquara. Biofilm thickness was performed by measuring the Z-stack of each biofilm using the ZEN BLUE 3.2 software.

**Fig. 1:**
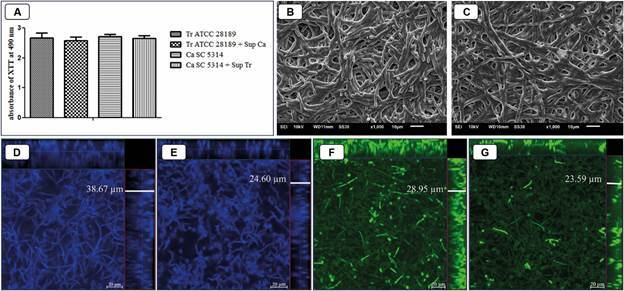
effect of cell-free supernatants from *Candida albicans* biofilms on *Trichophyton rubrum* biofilms and *T. rubrum* supernatant on *C. albicans* biofilms. Metabolic activity of biofilms measured by the XTT (2,3-Bis-(2-Methoxy-4-Nitro-5-Sulfophenyl)-2HTetrazolium-5-Carboxanilide) reduction assay (A). Scanning electron microscopy (SEM) images of *C. albicans* biofilms treated with cell-free supernatant from *T. rubrum* biofilms (B) and *T. rubrum* biofilm treated with cell-free supernatant from *C. albicans* biofilms (C). Confocal scanning microscopy of calcofluor white-stained *T. rubrum* biofilms without (D) or with cell-free supernatant from *C. albicans* biofilms (E). Confocal scanning microscopy of 5(6)-Carboxyfluorescein diacetate N-succinimidyl ester (CFSE)-stained of *C. albicans* biofilms without (F) or with cell-free supernatant from *T. rubrum* biofilms (G). Tr - *T. rubrum*; Ca - *C. albicans*; Sup - supernatant.

*Analysis of the expression profile of genes related to C. albicans filamentation by real-time polymerase chain reaction (RT-PCR)* - Samples from fungal biofilms directly cultivated were used for gene expression. As a control, monospecies biofilms of *C. albicans* were used. The material was collected, total RNA was extracted using the Illustra RNA spin Isolation Kit (GE Life Sciences), and the assay was performed as described by Costa-Orlandi et al.^9^ The concentration and purity of the samples were evaluated using the Nanodrop 2000 spectrophotometer (Thermo Scientific, Waltham, MA, USA) at absorbances of 260 and 280 nm. Integrity was assessed by capillary electrophoresis using the Agilent 2100 Bioanalyzer equipment (Agilent Technologies, Palo Alto, CA, USA). The relative expression was normalized using the primers ACT1 (F: GAAGCCCAATCCAAAAGA - R: CTTCTGGAGCAACTCTCAATTC), PMA1 (F: TTGCTTATGATAATGCTCCATACGA - R: TACCCCACAATCTTGGCAAGT). Specific primers to verify filamentation were designed: CPH1 (Forward: ACGCAGCCACAAGCTCTACT - Reverse: GTTGTGTGTGGAGGTTGCAC), HWP1 (F: GAAACCTCACCAATTGCTCCAG - R: GTAGAGACGACAGCACTAGATTCC), EFG1 (F: CAGTATGGTCAGTATAATGCT- R: TGTTGTTGCTGTTGGTATGGATATGATGATG), with efficiency between 91% - 119% [Supplementary data (Fig. 1)].^14^ ^,^ ^15^ The quantitative PCR (qPCRs) were performed using the 7500 Real-Time PCR Instrument (Applied Biosystems by Thermo Fisher Scientific) of the School of Pharmaceutical Sciences of UNESP - Araraquara with the Power SYBR™ Green PCR Master Mix detection system (Applied Biosystems by Thermo Fisher Scientific).

*Statistical analyses* - All data in this study are representative of three independent experiments performed in triplicates. The GraphPad Prism 5.0 software (GraphPad Software Inc., La Jolla, CA) was used for graph preparation and statistical analysis. P-values for multiple comparisons were calculated by one-way analysis of variance (ANOVA) and were adjusted by use of the Tukey’s post hoc analysis. P-values of < 0.05 were considered significant.

## RESULTS

*The supernatants reduced the thickness of the monospecies biofilms* - Cell-free supernatants of monospecies biofilms were not able to inhibit the metabolic activity of *C. albicans* and *T. rubrum* biofilm (Fig. 1A). The topographies of *T. rubrum* and *C. albicans* biofilms treated with the supernatants showed no structural and conformational differences compared to the untreated controls (Fig. 1B-C). In addition, the supernatant of *T. rubrum* biofilms did not reduce or prevent the filamentation of *C. albicans* (Fig. 1B). *T. rubrum* and *C. albicans* biofilms treated with the supernatants decreased in thickness by 36.5% (from 38.6 µm to 24.6 µm; p < 0.001; Fig. 1D-E) and 19.8% (28.93 µm to 23.2 µm; p < 0.01; Fig. 1F-G), respectively, suggesting that the microorganisms secrete molecules with the ability to interfere with biofilm formation mutually.

**Fig. 2:**
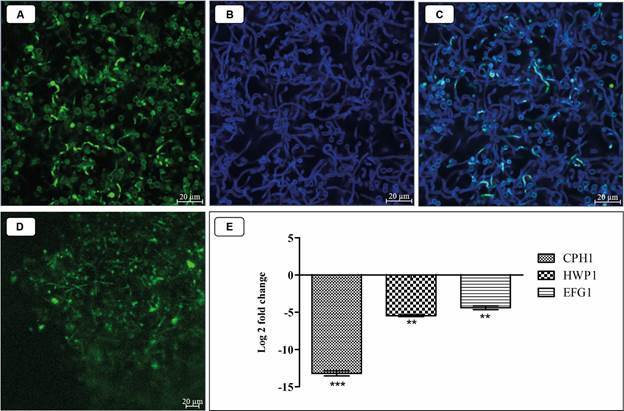
confocal scanning microscopy of *Candida albicans* cells (A) and *Trichophyton rubrum* cells (B) in mixed biofilm placed in direct contact. The merge of images in the mixed biofilm of *T. rubrum* and *C. albicans* (C). Biofilm of *C. albicans* indirectly separated with 0.4 µm pore transwell membranes to *T. rubrum* (D). The *C. albicans* cells was stained with 5(6)-Carboxyfluorescein diacetate N-succinimidyl ester (CFSE) and the *T. rubrum* cells was stained with calcofluor white. Relative expression levels of the transcriptional regulator involved in morphogenesis (CPH1), hyphal-specific protein (HWP1), and transcriptional regulator related to morphogenesis (EFG1), genes for the *C. albicans* SC 5314 strain in the mixed biofilms with *T. rubrum* represented in the Log2Fold (E). The results of two independent experiments are expressed as mean ± standard deviation (SD). Statistical significance was determined by Student’s T test and indicated by asterisks: ***p < 0.001, **p <0.01.

*Candida albicans filamentation is inhibited only in the direct interaction between C. albicans and T. rubrum in the polymicrobial biofilm* - The mixed biofilms of *C. albicans* and *T. rubrum* directly cultivated displayed many *C. albicans* yeast cells with minimal pseudohyphae, confirming the inhibition of the psudohyphal formation (Fig. 2A). Hyphae of *T. rubrum* were observed homogeneously interspersed with *C. albicans* cells without preference for a specific region of the biofilm (Fig. 2B-C). In contrast, *C. albicans* cells in indirectly cultivated biofilms showed significantly greater pseudohyphal formation (Fig. 2D). This result suggests that *C. albicans* probably need to interact directly with *T. rubrum* to modulate the molecules/pathways that inhibit yeast filamentation. The quantity of RNA in the samples ranged from 71.2 to 971.6 ng/µL, and the RNA integrity number (RIN) ranged from 5.90 to 6.90 [Supplementary data (Fig. 2)]. The relative expression of the adhesion gene (HWP1) and transcription factor regulatory genes (CPH1, EFG1) of *C. albicans* in mixed biofilms directly cultivated showed a significant negative modulation (p = 0.0039; p = 0.0014 and p = 0.0037) for all the genes tested (Fig. 2E). These results confirmed that the inhibition of *C. albicans* filamentation by *T. rubrum* in mixed biofilms may be related to downregulation of genes associated with filamentation.

## DISCUSSION

Garcia and collaborators^5^ have verified the antagonistic interaction of *T. rubrum* and *C. albicans*/*C. parapsilosis* biofilms *in vitro*. *Candida* species predominated in mixed biofilms with *T. rubrum*, but inhibited *C. albicans* filamentation, which is an important factor in this commensal’s virulence. This work evaluated the antibiofilm action of *C. albicans* and *T. rubrum* supernatants and the inhibition of *C. albicans* filamentation in the presence of *T. rubrum*. Our results indicate that cell-free supernatants could not inhibit metabolic activity or cause damage to the topographic structure of *C. albicans* and *T. rubrum* biofilms. However, the confocal microscopy images revealed a thickness reduction in the structure of both biofilms. The difference between the XTT assay and the confocal images may be related to the limitations of the XTT reduction assay, as nonlinearity between the number of microorganisms and the colorimetric signal has been widely reported.^16^ ^,^ ^17^

Recent studies have shown that the interaction of *C. albicans* with other non-*albicans* species (*Candida krusei* and *Candida glabrata*) or even bacterial species (*Lactobacillus rhamnosus*, *Lactobacillus casei*, and *Lactobacillus acidophilus)* caused a reduction in *C. albicans* biofilm-forming capacity in addition to inhibition of the transition of yeast cells to pseudohyphae.^18^ ^,^ ^19^ ^,^ ^20^ These results were observed when the *C. albicans* cells were cultured concomitantly with other microorganisms or treated with their supernatants. In addition, quorum sensing plays a crucial role in the secretion of bioactive molecules with inhibitory capacity towards other microorganisms, interfering with the development of biofilm elements, and cell morphology alterations.^13^ Our results also suggest that microbial supernatants may potentially have inhibitory activity on biofilm structural components such as the extracellular matrix. Quantifying biomass and the elements that make up the extracellular matrix may provide new insights into the dynamics of biofilms after treatments with microbial supernatants.

Previous studies have revealed that evaluation of the mixed biofilm, carried out by the simultaneous addition of both microorganisms, provides homogeneous initial conditions for the cells, this method tends to favor the predominant growth of *C. albicans*. This is due to the faster intrinsic metabolism of this microorganism compared to *T. rubrum*, resulting in a scarce presence of *T. rubrum* cells in the mature biofilm. On the other hand, the strategy of pre-adhesion of *T. rubrum* followed by the addition of *C. albicans* after an interval of 4 h minimizes the impact on the growth of both microorganisms in the mixed biofilm. This approach facilitates the analysis of cell behavior in the mixed biofilm.^5^ However, in this condition, *C. albicans* cells mostly bind and use *T. rubrum* as a substrate rather than the polystyrene surface, which may influence the modulation of adhesion patterns and other related processes. Furthermore, differences associated with the presence or absence of agitation during biofilm formation represent a considerable limitation when comparing monospecies and mixed biofilms.

Filamentation is a crucial phase of *C. albicans* pathogenicity, allowing the fungus to colonize and invade host tissues.^19^ To ensure visualization of the morphology of live and dead *C. albicans* cells, they were stained with CFSE before biofilm production. CFSE is a fluorophore that allows cell divisions to be monitored. Although the conditions of the *C. albicans* cells in the mixed and monospecies biofilms are not the same, the inhibition of filamentation of the *C. albicans* cells is noticeable in mixed biofilms directly cultured compared to those treated with the supernatants. Ribeiro and collaborators^18^ suggested that the presence of cells other than *C. albicans* in the multispecies biofilm may affect the growth of *C. albicans* cells due to increasing competition for nutrients and surface adhesion, among other factors. In addition, pseudohyphal formation by *C. albicans* can be inhibited by other microorganisms that secrete or have molecules in their cell wall responsible for negative filamentation signaling.^18^ ^,^ ^19^ ^,^ ^21^ Our findings suggest that *T. rubrum* inhibits important pathways in the transition of *C. albicans* yeast into pseudohyphae, and this competition may reduce this human commensal’s ability to colonize tissue and its pathogenicity. Negative HWP1, CPH1, and EFG1 gene modulation were observed in *C. albicans* cells from the mixed biofilm with *T. rubrum*. CPH1 and HWP1 regulate morphogenesis and control several cellular processes,^6^ and EFG1 is involved in *C. albicans* biofilm formation. It is known that a decrease in their expression impairs adhesion, invasion, biofilm formation, and, consequently, *C. albicans* virulence.^6^ ^,^ ^7^ ^,^ ^22^ ^,^ ^23^

*In conclusion* - The present work confirmed that the antagonistic interaction observed in the mixed biofilms of *T. rubrum* and *C. albicans* may be regulated by genes related to the filamentation of *C. albicans*, which may occur during interactions between both microorganisms in dermatomycoses. These findings warrant future studies that search for new molecules capable of inhibiting the expression of fungal virulence factors and can be used as effective therapeutics to combat prevalent dermatomycoses.
