# Supplementary material for: Trichophyton rubrum inhibits Candida albicans filamentation and its gene expression when grown in biofilms in vitro
Source: Mem Inst Oswaldo Cruz. 2025 Jun 27;120:e240221. doi: 10.1590/0074-02760240221 (PMC12208673; doi:10.1590/0074-02760240221)
Supplement: Supplementary file 1 [file 1678-8060-mioc-120-e240221-s.pdf]

A

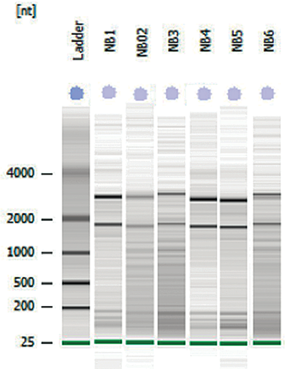

B

Number	Sample	A260/280 nm	ng/µL	RIN
**NB1**	*Candida albicans* - 1	2.23	971.6	6.60
**NB2**	*Candida albicans* - 2	2.28	661.0	5.80
**NB3**	*Candida albicans* -3	2.26	603.0	5.90
**NB4**	*Trichophyton rubrum* + *Candida albicans* 1	2.17	471.1	6.90
**NB5**	*Trichophyton rubrum* + *Candida albicans* 2	2.25	71.2	6.10
**NB6**	*Trichophyton rubrum* + *Candida albicans* 3	2.16	120.4	5.90

Fig. 1: primer validation for reverse transcription polymerase chain reaction (RT-PCR). A melting curve analysis was conducted to test the specificity of the primers CPH1, HWP1, EFG1, PMA-1 and ACT-1. The presence of a single peak is indicative of the existence of a single PCR product.

CC BY

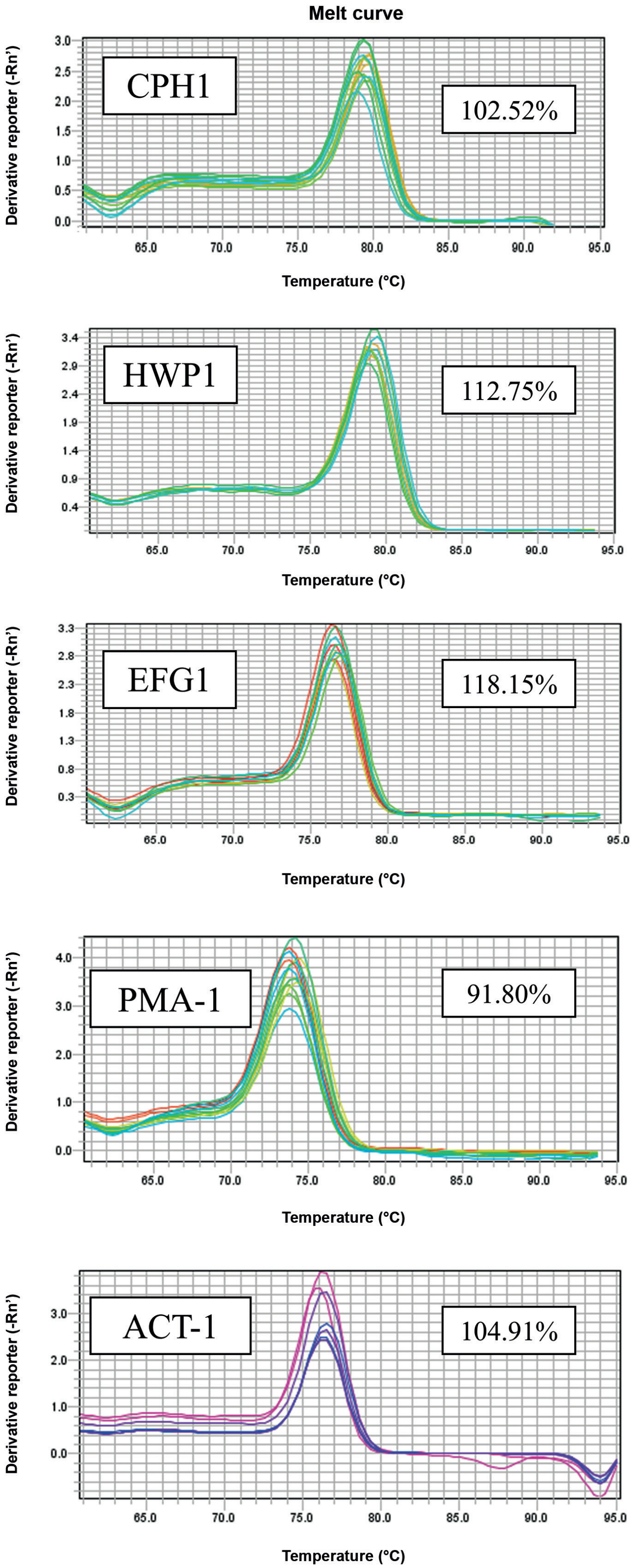

Fig. 2: capillary electrophoresis was conducted using the Agilent 2100 Bioanalyzer equipment (Agilent Technologies, Palo Alto, CA, USA). (A) The integrity of samples NB1, 2, 3, 4, 5, and 6 was validated. (B) The relationship between the ratio at A260/280 nm, concentration sample, and RNA integrity number (RIN) is shown.
